# A Functional Neuroimaging Analysis of the Trail Making Test-B: Implications for Clinical Application

**DOI:** 10.3233/BEN-2011-0278

**Published:** 2011-05-23

**Authors:** Mark D. Allen, Tyler E. Owens, Alina K. Fong, Douglas R. Richards

**Affiliations:** ^1^Psychology DepartmentBrigham Young UniversityProvoUTUSA; ^2^Neuroscience CenterBrigham Young UniversityProvoUTUSA; ^3^Rehabilitation UnitNeuropsychologyUtah Valley Regional Medical CenterProvoUTUSA

**Keywords:** fMRI, clinical fMRI, cognitive assessment, neuropsychological assessment, Trail Making

## Abstract

Recent progress has been made using fMRI as a clinical assessment tool, often employing analogues of traditional “paper and pencil” tests. The Trail Making Test (TMT), popular for years as a neuropsychological exam, has been largely ignored in the realm of neuroimaging, most likely because its physical format and administration does not lend itself to straightforward adaptation as an fMRI paradigm. Likewise, there is relatively more ambiguity about the neural systems associated with this test than many other tests of comparable clinical use. In this study, we describe an fMRI version of Trail Making Test-B (TMTB) that maintains the core functionality of the TMT while optimizing its use for both research and clinical settings. Subjects (*N* = 32) were administered the Functional Trail Making Test-B (f-TMTB). Brain region activations elicited by the f-TMTB were consistent with expectations given by prior TMT neurophysiological studies, including significant activations in the ventral and dorsal visual pathways and the medial pre-supplementary motor area. The f-TMTB was further evaluated for concurrent validity with the traditional TMTB using an additional sample of control subjects (*N* = 100). Together, these results support the f-TMTB as a viable neuroimaging adaptation of the TMT that is optimized to evoke maximally robust fMRI activation with minimal time and equipment requirements.

## 1. Introduction

Functional magnetic resonance imaging (fMRI) is becoming increasingly recognized for its potential in clinical applications [7,8,16]. This study represents a portion of a research project on the development of fMRI as a practical tool for the diagnosis and assessment of cognitive functioning, with the objective of producing fMRI tests that might be relied upon, in much the same manner as conventional "paper-pencil" neuropsychological evaluations are relied upon, as a routine form of cognitive assessment [32,33,39,45,59]. The work presented here concerns an fMRI adaptation of the conventional Trail Making Test (TMT) with specific focus on the latter of its two sub-tests, the Trail Making Test-B (TMTB). The resulting fMRI adapted protocol, which we refer to as the *f-TMTB* is one of several protocols developed as a comprehensive battery of fMRI assessments reported in recent literature. Previous reports include the *f-MRT*, an adaptation of the matrix reasoning test [2], the *f-VFT* and adaptation of the verbal fluency test [3] and the *f-PNT* [15] a picture naming test.

As discussed in the papers cited above, there is some consensus that the most fundamental requirements in developing clinical fMRI protocols, are that they should be standardized and validated (i.e., subjected to the same level of reliability and validity testing as any other new neuropsychological test) and that assessment outcomes from single individuals should be interpreted in the context of normative data (just as what is expected for the interpretation of any other neuropsychological exam). These issues are unique to clinical applications

ISSN 0953-4180/11/$27.50 © 2011 – IOS Press and the authors. All rights reserved of fMRI, as opposed to purely scientific research efforts, as the latter may typically rely on the power of group averaging, whereas the former must be able to produce meaningful fMRI data from a single individual (see [47]) for an especially sophisticated approach to this problem). In previous papers [2,3], we present full details about our approach for validity testing and acquiring normative data for fMRI assessment, such as the f-TMTB. A further important issue however, which is the focus of this paper, concerns the technical difficulties that confront attempts to create fMRI adaptations of familiar clinical neuropsychological tests. This comes from the fact that all paper-pencil neuropsychological tests were originally created without the need to accommodate the severe temporal, physical, physiological, and even financial limitations (both in research and clinical settings) imposed by the MRI scanning environment. In fact, the very activity perhaps most common to conventional exams – using a pencil and paper – is not possible in fMRI testing.

Despite these limitations, there are many examples of ingenuity, in which researchers have devised excellent adaptations of familiar neuropsychological tests, such as the Matrix Reasoning Test [39,48], the Verbal Fluency Test [6,45], the Hooper Visual Organization Test [33], the Wisconsin Card Sorting Task [21,23,32], the Tower of Hanoi Test [5,14], the Tower of London Test [12,35,36], and the TMT [30,59]. Although these studies represent particularly good examples of careful matching on crucial features of neuropsychological exams and their corresponding fMRI adaptations, exact protocol replications, in many cases, are simply not possible. Nevertheless, we suggest that valid fMRI adaptations need not always achieve exact replication. Rather, a good approximation using similar stimuli and response methods might well suffice, to the extent that the fMRI adaptation is empirically shown to measure the same cognitive construct that the conventional test is purported to measure. Thus, as described in Allen and Fong [2,3] concurrent validity testing might be used to evaluate new fMRI adaptations, in much the same way that new conventional tests are evaluated with respect to existing ones. With these issues at interest, we chose to focus on the traditional TMT in this study, because it appears to be one test that would seem especially difficult to accommodate for fMRI use.

The TMT has had an exceptionally long and popular history of use in cognitive assessment, as a measure of executive functioning, mental flexibility, psychomotor speed, and other lower- and higher-level cognitive processes. It originated as part of a U.S. Army testing battery, was later adopted into the Halstead-Reitan Battery [40], and is currently included in prominent handbooks of Clinical Neuropsychology [24,29,51]. The TMT is traditionally administered in two parts, where the Trail Making Test-A (TMTA) is administered first, which is assumed to tap lower-level perceptual/motor functions, and the TMTB is administered second, which is assumed to draw on additional higher-level cognitive mechanisms, along with the lower-level mechanisms it has in common with the TMTA.

Efforts to identify the neural substrates of the TMT, particularly the higher-level functions implicated in TMTB performance, have been undertaken using variety of neurophysiological techniques, including brain lesion-behavior mapping [4,17,41,52,53,58], scalp electroencephalography [45], repetitive transcranial magnetic stimulation [31], and near-infrared spectroscopy [57]. The above studies, however, have yielded some ambiguous results. For example, although one might assume that the TMTB would require prefrontal cortex involvement, it is not clear from these studies which prefrontal regions, if any, are necessary for successful TMTB performance. In light of these mixed findings, fMRI might offer an alternative source of neurophysiological evidence. To date, there are only two published fMRI studies that have been designed to examine the neural correlates of TMTB performance: Moll et al. [30] and Zakzanis, et al. [59]. Despite the fact that quite different implementations of the TMT task were used, these two studies yielded fairly similar results, with common activation in dorsolateral and medial prefrontal cortex (including supplementary motor area/dorsal anterior cingulate), and parietal cortex. In fact, the bulk of the differences between the two studies can be readily attributed to the fact that Moll et al. employed a verbal-response adaptation of the TMT.

The vastly different strategies employed by Moll et al. [30] versus Zakzanis et al. [59], described more fully below, highlights a major theme of this paper, which is the fact that there appears to be no straightforward way to directly modify the TMT for use in the MRI scanning environment, without compromising at least some critical feature of the task. According to standard administration of the TMT [40], the subject is presented with an array of numbers (TMTA) or an array of numbers and letters (TMTB) pseudorandomly distributed on a sheet of paper. With the starting point (the number 1) indicated, the subject is instructed to draw a continuous line connecting the remaining numbers in ascending sequential order (TMTA), or the remaining numbers and letters in alternating ascending sequential order (TMTB) as quickly and accurately as possible. According to standard administration, the subject is closely monitored during performance, and is provided with immediate error-feedback and error-correction, in which the administrator alerts the subject as quickly as possible to the error and physically returns the patient to the last correct position [40]. Because the primary performance measure on this task is total completion time, including administrator feedback time, it has been suggested that consistency is needed in establishing uniform feedback and correction procedures among test administrators [49].

Given the above description and administration requirements of the TMT, it becomes apparent that fMRI adaption raises significant technological design challenges, particularly when the objective of the fMRI design is to model the traditional protocol as closely as possible. One of the greatest limitations is that subjects must lie on their backs, keep their heads entirely still, and keep movement other extremities, such has hands and arms, to a minimum. Moreover, from the participant's point of view, it is difficult to see one's hands in this supine, fixed-head position in the first place. These factors, combined with the loud scanning environment also raise serious problems for implementing administrator feedback and manual error correction. Finally, standard administration of each TMT subtest is done in a single trial, where durations may range from 50 to 180 s on average for control subjects (e.g., for the TMTB [55]) and much longer for patients. This is not optimal for fMRI, where both empirical and simulation studies recommend that designs with more trials (e.g., 8) and shorter trial durations (e.g., 10–20 s) will greatly increase the paradigm's power, efficiency, and signal to noise resolution [19,25,26,28].

Moll et al. [30] is the first reported effort to address some of these problems. To do so, they relied on a verbal form of the TMT (vTMT) previously available as a behavioral paradigm [1], where subjects verbally produce number (vTMTA) or number-letter alternations (vTMTB) in ascending sequential order. For their fMRI adaptation, subjects performed this task covertly. Despite the many a priori differences in the hypothesized inventory of cognitive mechanisms required to perform the vTMT versus its standard form, the results obtained by Moll et al. were actually quite consistent with activation patterns one might expect for a cognitively demanding task of this sort, in addition to activation associated with language processing areas. A far superior solution, in our opinion, is described in Zakzanis et al. [59], with their innovative use of an MRI-compatible virtual stylus, which enabled subjects to draw connective lines on a supportive tablet and simultaneously view the action of their stylus movements on a projected image of the TMT. The receptive tablet was mounted within easy reach from their prone position on the scanner bed, such that excessive head motion was not induced. Activation found in this study, as summarized briefly above, fit remarkably well with what one would expect given the functional demands of the TMT. Perhaps the only shortcoming of the methods reported by Zakzanis et al., with regard to close modeling of the conventional administration of the TMT, was that subjects did not receive error feedback and exogenous correction. It is conceivable, though, that these features could be incorporated into their method with further refinements.

It should be kept in mind that the objectives of our study may differ from other similar studies in important respects. In common with previous studies, this is a hypothesis-testing study – we too wish to understand the neurological systems that are associated with performance of the TMT by comparing activation derived from our fMRI implementation to theoretical assumptions about the cognitive systems that the TMT engages. However, we are also operating under applied constraints, with the aim to produce a protocol that is optimized for use in real clinical radiological settings administered by non-cognitive neuroscientists. Because of this latter requirement, our protocol, and its accompanying system for normative data collection, must be capable of making valid and reliable conclusions about activation data from a single subject collected from a single relatively brief scanning session. As described here and elsewhere [2,3], single-subject interpretation is not possible in the first place without the context of normative data. However, both the normative data and the single-subject data are of little use if they are of poor quality or low construct validity. Thus, given our objectives to produce meaningful data at the single-subject level in a manner that can be implemented in real clinical settings, there is a considerable burden to create an optimized protocol that maximizes task-related signal for the cognitive mechanisms at interest that can be administered in the shortest amount of total scanning time possible, and with as little specialized peripheral apparatus as possible. Building on the work of Zakzanis et al. [59], then, we describe here the development and testing of the *f-TMTB*, including concurrent validity testing procedures and description of a method for evaluating task compliance. We also present a detailed analysis of functional activation at the group level and its relevance to the cognitive neuroscience of the TMT.

## 2. Materials and methods

### 2.1. Participants

Thirty-two participants (16 male, 16 female) between 20 and 39 years old (Mean = 25.04; S.D. = 4.23) volunteered as control subjects for this study. All participants gave their informed consent prior to inclusion in the study by reading a study description and signing a consent form approved by the Institutional Review Board of Brigham Young University. Participants received no compensation. Hand dominance was assessed using the Edinburgh Handedness Inventory [37]. All but two subjects (one male, one female) were determined to be dominantly right-handed. Mean L.Q. scores on the Edinburgh handedness scale – where scores above +48 suggest strong right handedness – were +71.8 (Decile R.3), S.D. = 35.0 for females; and +69.1 (Decile R.3), S.D. = 31.5 for males; with no significant difference between sexes ($t = 1.36$, $p > 0.1$). All participants spoke English as their first language. All participants were determined to have no history of neurological impairments (assessed by a screening questionnaire), nor history of significant psychological pathology, and reported no use of psychotropic medications. High resolution 3D SPGR and $T_2$ axial FLAIR MRI scans revealed no detectible brain abnormalities in any control subjects, as determined by a neuroradiologist. All subjects had completed at least one year of college education and were in good academic standing at a university with high admission/continuance standards. All participants consented to release pre-admission records of ACT (or SAT) scores. Analysis of mean scores (with SAT converted to ACT equivalents) revealed overall high performance, with a mean of 30 (S.D. = 4.30) for females, and 29 (S.D. = 2.16) for males, with no significant difference between sexes ($t = 1.38$, $p > 0.1$).

### 2.2. Apparatus and task design

The design of the f-TMTB is intended to accommodate research and clinical facilities with the most basic of peripheral equipment and software – requiring only devices for visual stimulus presentation and simple manual response collection. Additionally, to reduce total scanning time requirements, only the TMTB subtest is included (as the acronym suggests). Further rationale for this is presented in the discussion. Within these constraints, our solution employed a *partially covert response method*. The method was as follows: At the beginning of a trial, the participant viewed a starting image (Fig. 1a) displayed on a back-projection screen through angled mirrors mounted in the head coil. The start image displayed a pseudorandomly distributed array of 22 items, including the numbers 1 through 11 and letters A through K, with the number 1 circled in red. According to detailed instructions and examples received prior to entering the scanner, participants were instructed to first locate the circled number 1 and then make a visual search for the letter A. Upon locating the letter A, the subject was instructed to push a button on a fiber-optic response pad, where upon an arrowed-line was automatically drawn, connecting the number 1 to the letter A, and the letter A was circled in red (Fig. 1b). The arrow, then, served as both immediate feedback and correction. For example, suppose the subject had mistakenly identified the number 2 as the next target item. The arrow would then serve both to indicate that the subject had made a mistake, and to orient the subject back on the correct track, in a manner that approximates recommendations for standard administration [40]. Upon each button press, the previous connecting arrow disappeared, and the next linkage arrow was drawn (Fig. 1c). Once located, items remained circled throughout the remainder of the trial (Fig. 1d). On each trial, participants were given a time-limit of 22 s and encouraged to finish as many items as possible without making errors.

As seen in Fig. 1a-d, the horizontal layout of the f-TMTB fits as many items as possible within the approximate shape of the visual field one has while looking out through the scanner bore. Although standard TMT application leaves lines between previously connected items, pilot work indicated that, within the tighter horizontal "scanner-view" arrangement, leaving such lines intact was visually disruptive. However, in order to approximately maintain the cognitive demand effects of standard TMT application with its intact connections (i.e., that the search-set size progressively decreases), the f-TMTB leaves previously located items circled.

Another important issue concerns the covert nature of our design. One result of this is that, while subjects do get feedback when they make errors, we cannot actually record when and how often errors are made. Because the standard TMT scoring [40] does not actually tally errors per se, this is perhaps of less concern for our method than is a second issue, which is the problem of monitoring task compliance. However, we present below a promising solution that overcomes this limitation to a satisfactory degree.

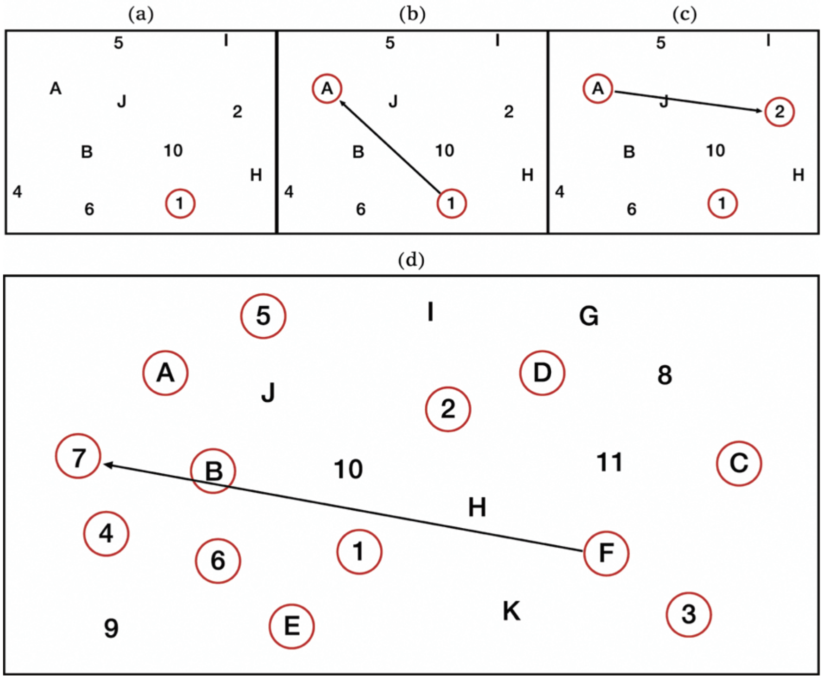

Fig. 1. On-screen appearance of the f-TMTB with examples of trial frames including the first three frames, (a, b, and c) and a latter frame (d). The first three frames are shown with a "zoomed-in" perspective.

### 2.3. *fMRI scanning procedure*

A total of 12 versions of TMTB test arrays were made to create two versions of the f-TMTB, each with 6 test epochs. Each test array employed a different pseudorandom arrangement of alphanumeric items (number 1 through letter K), with a different starting location. Prior to scanning sessions, participants were given detailed instructions and a chance to practice the computerized f-TMTB outside of the scanner. At the beginning of each session, a "please wait prompt" appeared for 8 s to allow for T1 relaxation effects. Each test epoch began with a 2-s "Get Ready" prompt, followed immediately by the start image (Fig. 1a), whereupon subjects were given 22 s to complete as many connections as possible via button presses as described above, using their right thumb. Each test epoch alternated with a 14-s "rest" epoch, in which subjects were instructed to count covertly from 1 to 10. This simple counting task is recommended as an optimal minimal-demand cognitive activity for rest epochs in fMRI experiments [50]. There were a total of 6 test-rest epoch cycles for a session duration of 4 min. After functional scanning, 3D SPGR and $T_2$ axial FLAIR images were acquired.

### 2.4. *Evaluation of concurrent validity: The f-TMTB and the standard Trail Making Test-B*

In order to assess correlations in performance between the f-TMTB and the standard TMTB [24,40], we collected additional data from a sample of 100 subjects without neurological or psychological impairment, who also gave informed consent to participate in this study. These additional control subjects were matched demographically to the participants in the fMRI study, in terms of age, sex, and education level. All participants completed both the standard TMTB and the computerized f-TMTB. Both tests were given in a single session with test-order counterbalanced across participants. Administration of the standard TMTB followed the instruction scripts, feedback/correction procedures, and timing procedures as outlined in Reitan and Wolfson [40]. The computerized f-TMTB was administered using the same instructions and practice procedures as applied to the fMRI participants. Performance on the standard TMTB was measured as total time to completion, whereas performance on the f-TMTB was measured as the average number of trials completed per test epoch, such that concurrent validity correlation had an expected negative direction. Results for this concurrent validity analysis are given in Section 4.1.1.1 below.

## 3. Data analysis

### 3.1. Image acquisition

Functional images were acquired with a 1.5-T GE scanner using an EPIBOLD sequence with the critical parameters TR = 2000 ms; TE = 40 ms; Flip Angle = 90. Images were acquired at 23 contiguous axial locations with a slice thickness of 5 mm, 0 mm interslice gap, with a 3.75 × 3.75 mm in-plane resolution and a 64 × 64 matrix of individual sample points, producing a total of 64 × 64 × 23 voxels for entire brain coverage. Preprocessing procedures included acquisition time realignment, using sinc interpolation, followed by motion correction with EPI distortion unwarping. No head movement exceeded 1 mm translation or 1° rotation displacement. After motion/distortion correction, all functional volumes were spatially normalized and resampled using the Montreal Neurological Institute (MNI) templates implemented in SPM5, and spatially smoothed with an 8mm FWHM Gaussian kernel, in order to increase signal-to-noise ratio and to reduce the effects of moderate intersubject variability in brain anatomy. A high-resolution 3D SPGR whole-head volume was also collected from each subject and examined by a neuroradiologist for any structural anomalies that might disqualify the participant as a "normal" control subject. Each subject's SPGR image was then coregistered and normalized to their mean functional image in order to perform subject-specific comprehensive ROI analyses that take into account individual variability in cortical landmark organization.

### 3.2. Conventional fMRI analyses

#### 3.2.1. Subject-level analysis

A time-series ANCOVA implemented in SPM5 was used to test each voxel, for each subject, against the null-hypothesis that changes in BOLD signal in that voxel, over the duration of the experiment, did not significantly correlate with the temporal sequencing of the test and rest epochs. A boxcar waveform convolved with a synthetic hemodynamic response function (HRF) with a 4 s lag-to-peak was used to model task-related activation. The data were high-passed-filtered in time, using a set of discrete cosine basis functions with a cut-off period of 128 s, and conditioned for temporal autocorrelations using AR1 correction. For each participant, $t$-values for the contrast test condition versus rest condition, as well as the simple contrast test condition (against an implicit baseline) were computed for each voxel, using the parameter estimates of the ANCOVA. The resulting 3-dimensional contrast map from each subject was saved for further subject-level ROI analysis as well as for random effects (RFX) group-level analysis.

#### 3.2.2. Group-level analysis

Activation at the group level was analyzed using the RFX approach recommended by Penny, Holmes, and Friston [38], in which the value of the sum of the contrast weights for each voxel from each subject's ANCOVA was entered as a single data point in a *second-level* $t$-statistic computation, with the mean value for each voxel across subjects modeled as the effect term and the variance between subjects modeled as the error term. Significant activation peaks at the group-level are reported with a critical family-wise error (FWE) corrected $p$-value of $< 0.001$, and a voxel cluster extent threshold of 8.

### 3.3. Comprehensive ROI analysis

In addition to the RFX group-level analysis, we performed comprehensive ROI-based analyses for each control participant for 48 functional brain regions, for each hemisphere (see [2,56]). This analysis was done primarily to derive normative data for the objectives of the overarching project within which this f-TMTB study was embedded. However, a second purpose of this custom analysis, beneficial to the objectives of the current study, is that is serves as an additional, complementary measure of reliable brain activation related to the TMTB, along with the RFX analysis. Specifically, the ROI analysis may be better suited to detect reliable activation of large functional areas (e.g., dorsolateral prefrontal cortex) where subjects may show reliable activation within the boundaries of that functionally-defined region, but with variable foci across subjects. Details for this custom ROI approach are given in Allen and Fong [2].

### 3.4. Analysis of task compliance

As described above, on each trial of the f-TMTB a subject makes a covert target search and then presses a button in order to verify correct target identification. This partially covert response method raises concerns about task compliance. For example, if a subject were not motivated to give genuine effort, s/he might simply push the button repeatedly until the experiment ends. However, because item-by-item response times

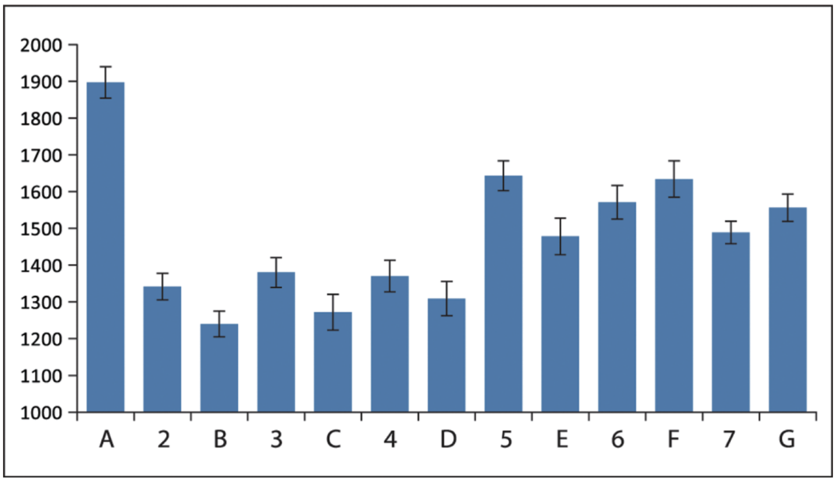

Fig. 2. Average reaction times (ms) and standard errors (+/-1) from 100 control subjects are plotted for the first thirteen target items of the fTMTB, A through G, showing an expected response profile for estimations of individual task compliance behaviour.

are logged, and because there are abrupt changes in task difficulty at designated points during the f-TMTB, task compliance can be determined with some confidence upon post-session analysis. The compliance-screening method we employ derives from analyses of item-by-item reaction-time profiles from the 100 control subjects who participated in the validity testing portion of this study. From these profiles, expected response characteristics related to task-difficulty changes were used to establish performance criteria that would allow estimates of task compliance for further individual subject/patients run on the f-TMTB. Results for this analysis are given in Section 4.1.1.2 below.

## 4. Results

### 4.1. Behavioral performance

#### 4.1.1. Results from analyses of additional 100 control subjects not participating in fMRI testing

##### 4.1.1.1. Results of concurrent validity analysis

For the standard TMTB, average completion time was 56.82 s (S.D. $= 16.72$). This is in good agreement with the norms provided by Tombaugh [55] for subjects in this age range and education level. The average number of completed trials on the f-TMTB was 17.09 (S.D. $= 3.27$), which did not differ significantly from the mean of the fMRI participants ($t < 1$), as reported in Section 4.1.2 below. Correlation analysis revealed a coefficient of $-0.69$, suggesting that the f-TMTB and standard TMTB show good concurrent/convergent validity.

##### 4.1.1.2. Results of task compliance analysis

In Fig. 2, mean response times are plotted for all subjects for each successive target item search from letter A (where number 1 is already circled at each trial start) through target letter G. This point, target G, was chosen as the end-point of analysis on the basis that it was the maximum point reached on 90% of all trials combined across all subjects. Maximum percentages dropped rapidly off after this point (e.g., 79% at the next target, 8, and 60% at the following target, H).

Inspection of Fig. 2 reveals several notable trends. First, there is obvious variance across trials. Moreover, the topography of variance across items is consistent across subjects, as indicated by error analysis (i.e., small standard error bars). Second, changes across trials correspond well with changes in task demands. To begin with, responses to target A are conspicuously longer than any other. This is easily explained by the fact that as the very first item, it would require some additional task start-up time, where, for example, the subject must first orient to the circled 1 and then search for the first target, A. Next, response times for the second half of items (5 through G) are notably longer than for the first half (2 through D, excluding initial target A). A reasonable explanation for this difference is that number/letter pairs 1-A, 2-B, 3-C, 4-D, are much more likely to be associated in declarative memory for most people than later pairs, such as 6-F. A related feature in this regard is the relative jump in response time from letter D to number 5. Finally, there is a clear number-letter latency alternation pattern for the first half of targets, where letters are responded to more quickly than their corresponding number associates. This might possibly

Table 1
Distribution of criteria violations by type for control subjects. Italics indicate criteria of special concern. Bold indicates 5 subjects showing violation patterns of special concern

| Criteria | | | | | | | | | | |
|---|---|---|---|---|---|---|---|---|---|---|
| *1. Overall Variance* | * | | | | | | | | | |
| *2. First Item Lag* | * | * | | | | | * | | | |
| 3. Split-Half Difference | * | * | * | * | * | | | | | |
| 4. Significant D-5 Jump | * | * | * | * | | * | | * | | |
| 5. Number-Letter Pattern | * | * | * | | * | * | | | * | |
| Number of controls/100 with violation pattern | **4** | **1** | 1 | 3 | 4 | 7 | 3 | 2 | 5 | 70 |

reflect an aspect of associative memory in which these number-letter items are stored as pairs, such that retrieval of the first item affords relatively fast access to its associate.

Given the well-characterized response features identified above, we may then form some empirically motivated criteria for estimating task compliance in individual subjects, where the fewer of these features there are in a subject's response profile, the less likely it is that the subject has shown good task compliance. However, one must also take into consideration atypical strategies for approaching this task that might affect response profiles. For example, if a subject were to happen to have number-letter pairs strongly associated in long term memory beyond the typical items 4-D, that subject might show a lack of split-half response difference and/or lack of jump from letter D to number 5. Thus, care should be taken to avoid categorizing an alternative strategy as poor compliance. Accordingly, we have formulated a rank-ordering of criteria importance, with features that can more reasonably be attributed to alternate task strategies (Items 3–5) at the lower end. The criteria order is listed below.

Compliance criteria:

1. Overall inter-item variance
2. First target response lag
3. Split-half difference (2 through D $<$ 5 through G)
4. Significant "jump" at D-5 (Connection D-5 $>$ Connection C-4)
5. Number-letter alternations for A through D

As mentioned above, it is reasonable to consider the first two criteria significantly more likely to indicate poor compliance than the remaining three. For example, a pattern that shows absolutely no variation in response times (violation of Item 1), particularly when those responses are uniformly quick, is not very likely to reflect an alternative task strategy. Likewise, it would be difficult to imagine lack of first item lag (Item 2) as reflecting some alternative response strategy. Thus we

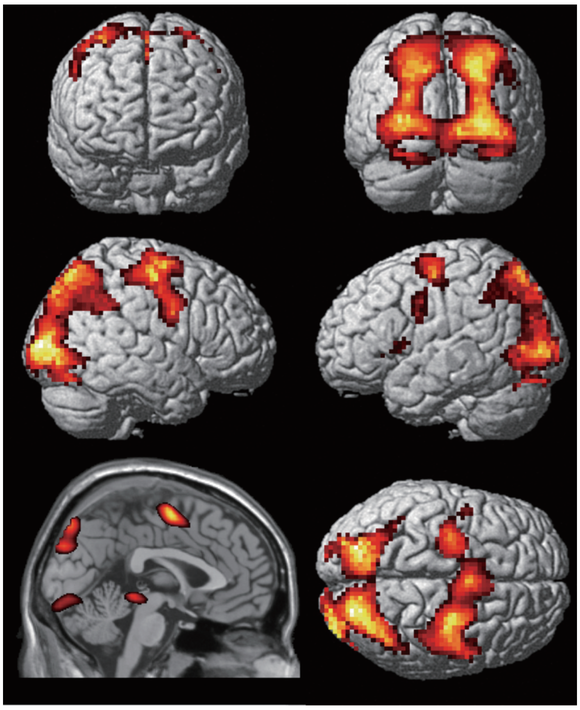

Fig. 3. Distribution of significant group-level RFX-modeled activation for the f-TMTB test. Rendered surface-projections and medial surface activation displayed on the single-subject MNI brain template in radiological convention (L = R).

suggest the following levels of confidence in compliance based on criteria violations: Violation of Item 1 (and by definition of all the others): *Low Confidence*; Violation of Items 2–5: *Moderate Confidence*; Any other combination of violations: *High Confidence*.

The criteria rankings and interpretations described above are further empirically motivated by analysis of criteria violations among the control subjects themselves. As displayed in Table 1, a total of 30/100 subjects showed criterion violations. Of these 30, there were 4 subjects with 5 violations; 1 with 4 violations; 1 with 3 violations; 14 with 2 violations; and 10 with 1 violation. Out of our control subject sample, then, we have low confidence that four subjects were com-

Table 2
Group-level random effects model activation foci ($p < 0.001$, FWE corrected) for the f-TMTB task

| Brain region | MNI Coordinates (x,y,z) | t- score |
|---|---|---|
| Middle Occipital Gyrus | | |
| Left | −26, −96, 5 | 14.79 |
| | −30, −85, 14 | 10.55 |
| Right | 27, −92, 6 | 13.99 |
| | 24, −83, 20 | 11.16 |
| Superior Occipital/Intraparietal Sulcus | | |
| Left | −25, −74, 39 | 11.11 |
| Right | 22, −76, 39 | 10.94 |
| Calcarine Cortex (V1) | | |
| Left | −10, −90, −1 | 8.11 |
| Right | 14, −78, 7 | 10.33 |
| Fusiform Gyrus/Inferior Occipital Gyrus | | |
| Left | −37, −73, −16 | 6.62 |
| Right | 26, −79, −14 | 5.80 |
| Premotor Cortex/Superior Frontal Gyrus | | |
| Left | −27, −9, 62 | 11.06 |
| Right | 29, −10, 59 | 8.77 |
| Precentral Gyrus | | |
| Left | −42, −7, 51 | 9.43 |
| | −47, −2, 29 | 8.51 |
| Superior Parietal Lobe/Intraparietal Sulcus | | |
| Left | −24, −69, 55 | 8.13 |
| Right | 23, −72, 56 | 9.94 |
| Medial Pre-Supplementary Motor Area | −1, 3, 57 | 9.08 |
| Midbrain | −2, −30, −48 | 7.12 |

pliant (these 4 subjects also showed unreasonably fast responses), and we have only moderate confidence that one further subject was compliant. Thus in all, our threshold suggests roughly 5% noncompliance. Data from the four participants with low confidence ratings were removed prior to computing the correlational analyses reported in the validity testing portion of this study. The consequences for doing this were negligible.

##### *4.1.1.3. Performance on the f-TMTB*

For the 32 control participants in the fMRI study, the mean number of completed trials (per test epoch) on the f-TMTB was 17.15 (S.D. = 3.88). We interpret this to reflect good performance, as it did not differ statistically from the mean of the 100 additional control subjects ($t < 1$), who in turn, showed good mean performance on the concurrently administered standard TMTB. No practice effects were evident across the 6 test epochs. We have reasonable confidence that all 32 fMRI participants were compliant with the task. Specifically, none of these participants showed violations of any more than 2 compliance criteria, as described in Section 4.1.1.2.

### *4.2. Group-level BOLD activation: RFX model*

Outcomes for group-level analyses based on the contrast test-rest (rest = counting task) did not differ in any notable way from the simple contrast test (versus implicit baseline). Areas of suprathreshold activation for the latter contrast are displayed in Fig. 3 and summarized in Table 2. Several peaks of activation with large cluster extents were found bilaterally throughout the ventral and dorsal visual processing streams, extending from fusiform cortex, through inferior and middle occipital cortex, reaching the parietal lobe with foci in intraparietal sulcus. Significant bilateral precentral gyrus/premotor activation is also present, with greater prominence on the left. Additional peaks of activation were present in medial pre-supplementary motor area and the tectal area of the midbrain.

### *4.3. Comprehensive ROI analysis*

According to the method described in Allen and Fong [2], independent activation peak values were extracted from customized ROI parcellation maps of each subject's brain and structured into a normative database. Furthermore, for the purposes of the current study, the comprehensive ROI analysis served as a complementary approach to the RFX analysis. Compared with previous studies from this research project [2,3] the results of the ROI analyses in this study were relatively less divergent from those of the RFX analysis. However, the ROI analysis did reveal two additional regions of reliable activation across subjects missed by the RFX analysis, including bilateral superior and middle frontal gyrus in regions anterior to the RFX peaks, as well as bilateral inferior frontal gyrus and anterior insula. These frontal peaks, though significant at comparable thresholds of the RFX model, were relatively small and found in variable foci across subjects. This is perhaps explanatory of inconsistent findings regarding necessary prefrontal involvement in the TMTB using fMRI and other methodologies. Further discussion of minor ROI/RFX outcome differences is omitted for brevity.

## 5. Discussion

Group-level activation for the f-TMTB in this study was consistent with expectations from previous studies using neuroimaging and other methods [4,17,30,31,45, 52,53,57–59]. Consistent with the dominant visuospatial nature of this task, with no subtraction task included, strong clusters of activation were found throughout the ventral and dorsal visual processing streams, extending from fusiform cortex, through lateral portions of inferior and middle occipital cortex, extending into parietal lobes, with foci in intraparietal sulcus. Bilateral precentral gyrus/premotor activation is also present, where greater activation on the left is most readily attributed to manual motor responses, but also includes activation on the right. This is consistent with studies demonstrating activation in bilateral precentral/premotor areas for tasks involving cognitive spatial processing over and beyond manual motor response demands [22,42].

Other notable regions of activation include medial pre-supplementary motor area (pSMA) and midbrain in the area of the colliculi. The pSMA is increasingly recognized for its role, along with adjacent medial prefrontal structures, such as the dorsal anterior cingulate, in the performance of demanding tasks such as the TMTB, in terms of response control, performance monitoring, error detection, feedback, uncertainty, response inhibition and related processes [10,18,34,54]. The peak of activation in the pSMA is within a region identified as the Rostral Cingulate Zone [43,44], which overlaps with anatomical loci labeled "dorsal anterior cingulate" in other studies on cognitive control and performance monitoring [9,11,20,27]. Given that the TMT relies heavily on visual-spatial search mechanisms, one would expect activation of the superior colliculus, which was clearly present in this study (see Fig. 2). The fact that activation was detected in this small, subcortical structure, where scanning parameters were optimized for full-brain coverage, gives us confidence in the effectiveness of our paradigm in eliciting clean, valid, and robust activations. Finally, we note that there was very little dorsolateral prefrontal activation, anterior to pre-motor areas. Although our ROI analysis revealed at least one significant area of activation in superior and/or middle frontal gyrus in all 32 subjects, exact loci were not consistent enough across subjects to result in suprathreshold peaks on the group-level RFX model. This outcome appears consistent with ambiguous findings for the role of prefrontal cortex in TMT performance, as discussed above.

An obvious feature of the f-TMTB study presented here, compared with previous studies – particularly the one with the design closest to ours – Zakzanis et al. [59], is that we did not include a TMTA task as a subtraction condition. The decision to not include the TMTA task is the simple result of a cost/benefit evaluation where the value of the information gained is pitted against the resources required to obtain that information. In our opinion, the amount of useful information gained by collecting brain activation associated solely with the TMTA is not worth the extra time it would take to include that condition in the protocol. By doing so, the f-TMTB is optimized for clinical application both in Neuropsychology, where primary interest is typically on the TMTB in the first place, not the TMTA, and in Radiology, where there are severe demands for protocols that yield maximum diagnostic information in the shortest scan time. To put this in perspective, the f-TMTB, with only six repetitions of the activation task, lasts about 4 min. In the context of a "full" neuropsychological evaluation of a patient, which is what is typically requested in our experience, the f-TMTB is likely to be only one of several assessments to administered in a single scanning session, with additional time needed for patient and scanner set-up, instructions, structural scan acquisition, etc.

The f-TMTB employs a partially-covert response method. While this solution requires minimal peripheral fMRI equipment, it also requires a special solution to verify task compliance. In this paper, we have dedicated a considerable amount of space describing details of this solution. One should not get the impression from this, however, that compliance monitoring for the f-TMTB is complicated and time-consuming. To date, the f-TMTB and its accompanying compliance metric has been used in fMRI assessments of over 100 patients with a wide variety of neurocognitive impairments. In most cases, a brief examination of the session log file upon immediate completion of the test is sufficient to verify task compliance.

Given the design differences between our approach and the closely related approach of Zakzanis et al. [59], a closer look at activation differences between the studies is in order. It would appear that the bulk of the differences between the two studies can be attributed to the omission of the TMTA subtraction task in our study. In particular, our results show robust activation throughout the entire ventral and dorsal visual processing systems. These additional areas of activation in our study, however, would not appear to obscure alternative activation findings had a subtraction been included, given that these visual path activation areas do not overlap with any regions identified in the TMTB-TMTA contrast of Zakzanis et al. Second, concerning the important issue of prefrontal cortex involvement, Zakzanis et al. found activation in superior and middle frontal gyrus, anterior to premotor areas, particularly in the left hemisphere, as well as bilateral insula. Our ROI analyses, though not reported in detail here, also revealed at least one peak of activation in middle and superior frontal gyrus in 32/32 subjects, with an overall greater likelihood of occurring on the left, but with variable foci throughout these large prefrontal regions across subjects. Likewise, these ROI analyses revealed reliable insula activation across subjects, but in regions more anterior to those found by Zakzanis et al., and tended to include inferior frontal gyrus as well. Finally, we note that in general, the activation peaks of Zakzanis et al. are substantially lower than ours in terms of $t$-values, with many functionally important foci falling well within white matter regions and/or including large cluster extents predominantly within white matter. While it is possible that these two factors might reflect the use of a subtraction task, with respect to divergence from our findings (i.e., larger $t$-values, activation restricted to grey matter), it is also possible that it reflects sample size differences, where our study included more than double the number of subjects (32 versus 12).

The primary focus of this study concerns the clinical implications of our findings. The TMT is among the very oldest neuropsychological tests in common usage. Nevertheless, relatively little work has been done in verifying assumptions about the neural systems that underlie this task, where the lack of functional neuroimaging work is especially notable. For example, compare the fact that there have only been 2 published fMRI studies to date on the TMT, while studies on other common neuropsychological tests, such as verbal fluency, abound in the literature (see, for example, Costafreda et al. [13] for a review of 22 recent fMRI studies on verbal fluency). A likely reason for this is that a task like verbal fluency is relatively easy to adapt for fMRI use than a task like the TMT. We have presented here an adaptation of the TMTB which is designed to engage all of the primary cognitive components of this classic test within the limits of the fMRI scanning environment. Using a large sample size and a careful design, we obtained robust activation in brain regions hypothesized to be central to the TMTB with minimal scanning time requirements. Thus, we present the f-TMTB (along with its accompanying assessments [2,3]) to both research and clinical users and encourage its use as a standardized cognitive fMRI assessment. We maintain that the f-TMTB meets critical requirements for clinical use as proposed in the introduction—namely, that clinical fMRI protocols should be standardized and validated; that data from individual patients should be interpreted in the context of normative data; that protocols should be designed to yield clean and robust activation from single subjects/patients; that technological barriers should be overcome, such that the fMRI protocol approximate its familiar counterpart(s) to every extent possible; and that concurrent validity testing be performed with respect to such traditional assessments.

## Acknowledgments

The authors express appreciation to Dr. Wendell Gibby M.D., of the Riverwoods Advanced Diagnostic Imaging Center, Provo, UT, for generous MRI facility support for this project.
